# What justifies public engagement in health financing decisions?

**DOI:** 10.2471/BLT.24.291860

**Published:** 2025-01-01

**Authors:** Matthew S McCoy, Johan L Dellgren, Ezekiel J Emanuel

**Affiliations:** a Department of Medical Ethics and Health Policy, Perelman School of Medicine, University of Pennsylvania, Blockley Hall, 423 Guardian Drive, Philadelphia, PA 19104, United States of America.

## Abstract

The World Bank’s report, *Open and inclusive: fair processes for financing universal health coverage,* represents an important effort to specify the benefits and criteria of fair processes in health financing decisions. Here we argue that the report’s justification for increasing public engagement in health financing decisions, one of its most novel contributions, rests on a widely shared but flawed assumption that public engagement will produce more equitable outcomes. Examining evidence from national-level public engagement initiatives cited in the report, we argue that there is no reason to assume that engaged publics will prioritize equity over other relevant values such as the maximization of population health. We conclude that instead of seeing public engagement as a tool for advancing particular values, policy-makers should view it as a neutral way of assessing what the public values and gathering insights that can inform the design of health benefits packages. If policy-makers wish to prioritize equity, they should do so directly through substantive policy choices regarding the design and financing of coverage schemes.

## Résumé

Le rapport de la Banque mondiale, intitulé* Ouverts et inclusifs: processus de financement équitables de la couverture sanitaire universelle,* représente un effort majeur dans la définition des avantages et critères propres aux processus de décision équitables pour le financement de la santé. Dans le présent document, nous expliquons pourquoi l'une des contributions les plus novatrices du rapport, à savoir l'argument selon lequel il faudrait que le public participe davantage aux décisions de financement en matière de santé, repose sur une hypothèse largement répandue mais néanmoins erronée selon laquelle ce type de participation produirait des résultats plus justes. Après avoir examiné les faits cités dans ce rapport, nous estimons qu'il n'y a aucune raison d'affirmer que les publics impliqués privilégieront l'équité aux dépens d'autres valeurs pertinentes telles que l'amélioration de la santé au sein de la population. Nous concluons qu'au lieu de considérer la participation du public comme un outil servant à faire progresser certaines valeurs en particulier, les responsables politiques devraient la voir comme un moyen neutre de déterminer ce qui est important aux yeux du public, et de récolter des informations utiles à l'élaboration d'avantages sociaux dans le domaine de la santé. S'ils souhaitent favoriser l'équité, ils doivent le faire directement au travers de choix politiques de fond concernant l'élaboration et le financement de régimes de couverture.

## Resumen

El informe del Banco Mundial, *Abierto e inclusivo: procesos justos para financiar la cobertura sanitaria universal*, representa un esfuerzo importante por especificar los beneficios y criterios de los procesos justos en las decisiones de financiación sanitaria. Aquí se argumenta que la justificación del informe para aumentar la participación pública en las decisiones de financiación sanitaria, una de sus contribuciones más novedosas, descansa en una suposición ampliamente compartida pero errónea de que la participación pública producirá resultados más equitativos. Tras examinar los datos de las iniciativas nacionales de participación pública citadas en el informe, se argumenta que no hay razón para suponer que los ciudadanos comprometidos priorizarán la equidad sobre otros valores relevantes, como la optimización de la salud de la población. Se concluye que, en lugar de ver el compromiso público como una herramienta para promover valores particulares, los responsables de formular las políticas deberían verlo como una forma neutral de evaluar lo que valora el público y recopilar información que pueda servir de base para el diseño de paquetes de beneficios sanitarios. Si los responsables de formular las políticas desean dar prioridad a la equidad, deberían hacerlo directamente a través de opciones normativas sustantivas relativas al diseño y la financiación de los sistemas de cobertura.

## ملخص

إن تقرير البنك الدولي بعنوان الانفتاح والشمول: العمليات العادلة لتمويل التغطية الصحية الشاملة، إنما يمثل جهدًا مهمًا لتحديد فوائد ومعايير العمليات العادلة في قرارات تمويل الصحة. وهنا نزعم أن تبرير التقرير لزيادة المشاركة العامة في قرارات تمويل الصحة، وهي واحدة من أحدث مساهماته، يستند إلى افتراض مشترك على نطاق واسع ولكنه غير صائب، وهو أن المشاركة العامة ستؤدي إلى نتائج أكثر إنصافًا. وعند فحص الأدلة من مبادرات المشاركة العامة على المستوى الوطني الواردة في التقرير، نزعم أنه لا يوجد سبب للافتراض بأن الجمهور المشارك سيمنح الأولوية للإنصاف فوق القيم الأخرى ذات الصلة، مثل تعظيم صحة السكان. ونخلص إلى الاستنتاج بأنه بدلاً من اعتبار المشاركة العامة كأداة للارتقاء بقيم معينة، يجب على واضعي السياسات أن يعتبرونها كطريقة محايدة لتقييم ما يقدره الجمهور، وجمع الأفكار التي يمكن أن تعود بالنفع على تصميم حزم الفوائد الصحية. وإذا أراد واضعو السياسات منح الأولوية للإنصاف، فيجب عليهم القيام بذلك بشكل مباشر من خلال خيارات سياسية جوهرية فيما يتعلق بتصميم مخططات التغطية وتمويلها.

## 摘要

世界银行发布了《开放和包容：为全民健康覆盖筹资的公平程序》，该报告重点强调了在卫生筹资决策方面采用公平程序的益处和标准。我们在此认为，该报告鼓励在卫生筹资决策方面扩大公众参与度的原因是基于一个广泛分享但有缺陷的假设，即公众参与有利于达成更加公平的决策，这也是世界银行提出的最新理念之一。通过研究报告中引用的国家级公众参与倡议相关证据，我们认为参与的公众不会将公平问题置于其他相关价值观（如最大程度地提高人群健康水平）之上予以优先考虑。研究结论表明，政策制定者不应将公众参与视为推进特定价值观的工具，而应将其视为一种评估公众关注点和收集见解以为健康福利方案的设计提供信息的中立方式。如果政策制定者希望优先考虑公平问题，他们应该直接要求就健康覆盖计划的设计和筹资开展实质性政策选择来实现这一点。

## Резюме

Доклад Всемирного банка *«Открытость и инклюзивность: справедливые процессы для финансирования всеобщего охвата услугами здравоохранения»* представляет собой важную меру, направленную на определение преимуществ и критериев справедливых процессов при принятии решений о финансировании здравоохранения. В данном отчете утверждается, что обоснование необходимости расширения участия общественности в принятии решений о финансировании здравоохранения является одним из его наиболее новых вкладов и основывается на широко распространенном, но ошибочном предположении, что участие общественности приведет к более справедливым результатам. В соответствии с данными, полученными в ходе реализации инициатив по вовлечению общественности на национальном уровне, приведенных в докладе, утверждается, что оснований полагать, что вовлеченная общественность будет отдавать предпочтение справедливости перед другими значимыми ценностями, такими как максимальное улучшение здоровья населения, нет. Сделан вывод о целесообразности рассматривать вовлечение общественности не как инструмент отстаивания определенных ценностей, а как нейтральный способ оценки общественных ценностей и получения информации, которая может послужить основой для разработки пакетов медицинских услуг. Если ответственные лица стремятся поставить справедливость в качестве приоритета, им следует сделать это непосредственно через выбор основных направлений политики, касающихся разработки и финансирования программ страхования.

## Introduction

Achieving universal health coverage (UHC) requires making difficult decisions. Policy-makers must determine how to mobilize revenue to finance health coverage schemes, who should be covered using pooled funds, and – most difficult of all – which products and services should be included in essential health benefits packages with what out-of-pocket payments.^1^

Ethical theory rarely dictates one right answer to these questions. Instead, there are a range of ethically acceptable answers, each of which strikes a different balance between competing values and interests, creating different winners and losers. Confronted with these complexities, many have argued that policy-makers should adopt fair decision-making processes for UHC – so that even those who would have preferred different outcomes can recognize the ultimate decisions as fair. Indeed, international organizations, government agencies, nongovernmental organizations and others have routinely affirmed the need to establish fair processes for health financing decisions.^2^^–^^5^

However, uncertainty remains about what a fair process entails and what objectives it should accomplish. The World Bank’s *Open and inclusive: fair processes for financing universal health coverage* report^1^ (the *Open and inclusive* report) is the latest effort to provide answers to these questions. Given the World Bank’s central role in financing and advising health systems throughout the world, policy-makers in countries striving to achieve UHC will likely consider the report authoritative.

Despite the report’s strengths, its justification for increasing public engagement in health financing decisions, one of its key contributions, rests on flawed assumptions. Because these assumptions are widely shared, analysing them could help prevent future problems with public engagement. The report proposes public engagement as a mechanism for advancing health equity. This proposal amplifies similar arguments in peer-reviewed literature^6^^,^^7^ and prior United Nations and World Bank reports claiming that engaging the public in resource allocation decisions will produce more equitable outcomes.^8^^–^^10^ This claim is mistaken. Instead of seeing public engagement as a tool for advancing particular values such as health equity, policy-makers should view it as a neutral way of assessing what the public values and gathering insights that can inform the design of health benefits packages.

## Benefits of fair processes

According to the *Open and inclusive* report, fair processes have four key benefits. They can: (i) “contribute to more equitable outcomes because they ensure that steps are taken to address common sources of inequity”, particularly the under-representation of poor and marginalized groups in decision-making; (ii) “strengthen the legitimacy of a decision process, which generally refers to the level of acceptance people have towards the authority of the government and of a polity’s laws and institutions”; (iii) “help build trust in public institutions by treating people affected by decisions with respect, explaining the underlying core rationale for the decisions, and ensuring that all affected constituencies are heard, with no group’s interests misrepresented or neglected”; (iv) “promote the implementation and sustainability of adopted policies” by generating “broad popular support even under conditions of disagreement”.^1^

Importantly, legitimacy, trust and sustainability are second-order benefits in that their significance stems from the indirect influence of fair procedures on public opinion. Only equitable outcomes are a first-order benefit. Such outcomes arise from the direct results of decision-making processes – not how they are publicly perceived. While the concept of health equity is open to varying interpretations, the sense of equitable outcomes intended by the report seems to be outcomes that give special priority to a society’s worst-off and ensure that “all people receive the health services they need without financial hardship”.^1^

## The criteria of fair process

How do fair processes contribute to achieving equitable outcomes? The *Open and inclusive* report presents several criteria for fair processes, organized in three domains: information, oversight and voice.

Information requires that a government provides transparent and accurate information about its decisions, and that it explains those decisions in terms that citizens can understand and accept. Oversight involves establishing mechanisms to ensure that health financing decisions are implemented effectively, while also allowing for the review, challenge and revision of these decisions when new evidence or arguments emerge. According to the report, voice requires “creating opportunities for the public to directly participate in the decision-making process and influence the outcome”, and mechanisms “for bringing in voices that typically would not contribute to public policy and decision-making”.^1^

While important, the criteria included in the information and oversight domains are uncontroversial. Indeed, they are similar to criteria included in previous accounts of procedural fairness, most notably the Accountability for Reasonableness framework.^11^ Conversely, voice, and its requirement for direct and influential public participation in health financing decisions, is novel. The voice criteria explicitly go beyond the requirements of the Accountability for Reasonableness framework, which, the report notes, has been criticized for placing insufficient emphasis on public participation.^1^

Of the three domains, voice is also the most directly related to the goal of promoting equitable outcomes:

“*a key source of inequity is power differences among stakeholders, which can lead to powerful stakeholders shaping the decision process to suit their own interests, [whereas] broadening participation and representation in the decision-making…can contribute to leveling the playing field towards greater equity*.”^1^

Thus, while the World Bank’s account of the benefits and criteria of procedural fairness is complex, its justification for public engagement rests on the assumption that engagement will advance equity as a guiding value in health financing decisions.

This assumption also informs the report’s discussion of the appropriate levels of public participation in different health financing decisions. The World Bank argues that public participation is typically most appropriate “in decisions that set the key directions for health financing”, such as which values should be affirmed and prioritized in the establishment of national insurance schemes.^1^ The report describes Peru’s adoption of the *Acuerdo Nacional*, which “affirmed the goal of ensuring universal access to health-care services and social security”, as an example of this type of directional decision. By contrast, the report describes Peru’s “decisions on design and allocation of funding to various budgetary programmes (e.g. nutrition, maternal and neonatal health, cancer prevention and control)” as technical and appropriately driven by experts and government officials. By engaging the public in directional decisions, the report suggests, countries can increase the likelihood that such decisions will prioritize health equity as opposed to the interests of powerful stakeholders. 

## Public engagement and equity 

The problem with positioning public engagement as a means of advancing health equity is that there is no reason to assume that the public will prioritize equity over other relevant values such as health maximization. Indeed, in multiple instances of national-level public engagement cited by the World Bank, members of the public appeared to prioritize other values over reducing inequities. For example, Israel’s Health Parliament did a public consultation by recruiting members of the public based on a stratified random sample of 1500 people from the adult population with an over-sampling of minorities and new-immigrant populations.^12^ Ultimately, organizers divided the participants into six groups to deliberate on health policy questions that raised equity issues. When asked whether people should be allowed to pay to ensure their choice of doctor in a publicly-funded hospital, four of six groups answered yes, reasoning that while allowing financially better-off citizens to pay for premier physicians could create inequities, it would increase overall revenue flowing into the health system. Similarly, asked whether co-payments for medical services provided under Israel’s National Health Insurance system should be maintained, five of the six groups answered yes, reasoning that while co-payments could pose access barriers for those worse-off, they would help to finance the health-care system. Across both questions, observers concluded, participants felt “responsibility to find ways to secure funds for the public health-care system, even if this infringed on equity considerations”.^12^

In another case cited by the World Bank, 514 Chileans were randomly selected to participate in a deliberative poll intended to gauge public opinion on policy proposals for Chilean pension and health-care systems.^13^ Participants were polled before and after a three-day deliberation, which included reading material and discussions with experts. Before deliberation, participants were generally supportive of increasing access to medical care for people with rare diseases, and for funding a national plan to train specialists to treat those with rare diseases. However, after deliberation, less than half agreed to increase payroll taxes to “guarantee access, funding, treatment and care for rare diseases” or “fund a national plan for the training of specialists”. Notably, these programmes would have been funded by increases in payroll taxes, not by a reallocation of existing public health funds, so they would have had no impact on access and quality of primary care.

These examples highlight a tension between competing ethical commitments in the *Open and inclusive* report’s account of public engagement. Justifying public engagement on the grounds that it contributes to equitable outcomes assumes a substantive commitment to prioritizing equity in health financing decisions. Yet, the idea that fair processes should create opportunities for the public to directly participate in the decision-making process and influence the outcome assumes a procedural commitment to responsiveness to public values. But, as Israel’s Health Parliament example shows, even oversampling from marginalized groups does not ensure that engaged publics will prioritize equity over other values. Additionally, as the Chilean example demonstrates, access to information and structured deliberation do not necessarily make engaged publics more likely to support expanding access to care.

These examples do not imply that engaged publics will never prioritize equity over other values. But they highlight the fact that publics hold diverse values and, therefore, that a sincere commitment to public engagement may not align with the goal of promoting equitable outcomes or any other particular goal that policy-makers have.

## Implications

These observations suggest several implications for equity and public deliberation. First, if policy-makers wish to prioritize equity, they should do so directly through substantive policy choices regarding the design and financing of coverage schemes, and publicly justify those choices. They should not do so surreptitiously, assuming that engaged publics will necessarily assign high priority to equity relative to other values.

One might argue that, in certain contexts, policy-makers who wish to prioritize equity in health financing decisions will be more successful if their efforts are supported by a mandate from engaged publics, and that policy-makers should therefore leverage public engagement to achieve their predetermined policy goals. However, aside from the potential for such a strategy to backfire, engaging the public with the intention of producing a desired outcome risks reducing public engagement to the kind of performative, tokenistic exercise the report warns against.^1^

Second, this critique of the World Bank’s report does not imply that there is no justification for public engagement in health financing decisions. Public engagement could generate legitimacy, trust and sustainability benefits. However, since these benefits depend on public perceptions, the extent to which they are realized may vary from country to country. In countries with a strong tradition of public involvement, increasing public participation in health financing decisions may improve legitimacy, trust and sustainability. In countries without such a tradition, these benefits may be less certain. The report does provide evidence that fair processes have improved legitimacy, trust and sustainability in certain settings. But as noted, fair processes are multifaceted, and it is not clear which of their many aspects are responsible for producing these benefits. Future research should examine the extent to which public participation uniquely contributes to legitimacy, trust and sustainability.

Ultimately, there are more straightforward justifications for public engagement. One reason to engage the public in health financing decisions is simply to understand what the public values, so that policy decisions can be better aligned with public priorities. Democratic countries already have representative institutions in place that are designed to channel public preferences into the policy-making process. However, processes like elections are ill-suited to capturing public views on specific health policy issues, particularly in technical areas such as health system financing. The manner in which the outputs of direct engagement mechanisms like deliberative polls and citizen juries ought to be integrated with existing democratic processes is a complicated issue, which we cannot explore here.^14^ Nevertheless, it is reasonable to think that across a range of ethically acceptable choices, policy-makers ought to favour options that align with public values, and that well-designed public engagement mechanisms might contribute to this goal.

This approach to engagement is best suited to addressing directional questions, as suggested in the World Bank’s report. However, such questions do not need to be limited to abstract values; they can also address more concrete matters such as the design of essential benefits packages. In either case, it is critical that engaged members of the public are representative of the relevant population, so that their judgements function as a reasonable proxy for the public’s values, and are not selectively chosen to achieve a desired outcome.

Another reason to involve the public in health financing decisions relies on the informational benefits of public engagement. By virtue of their experiences as patients, parents, caregivers and workers, lay citizens often have insights that can inform the design of health-care institutions and services.^15^ Individuals in rural communities might, for example, speak to the impact of rural hospital closures. New and expectant mothers may offer important perspectives on essential prenatal services. In such cases, the goal of engagement is not to gauge public values, but to gather firsthand accounts of relevant facts that can inform policy decisions. This approach to engagement is suited to more technical questions, where public insights offer additional evidence that can shape policy solutions. In this context, rather than recruiting for representation, policy-makers should seek to engage those most likely to use the services in question. In addition to informational benefits, this approach to engagement also demonstrates respect for persons by taking seriously their perspectives on how services can be designed to best meet their needs.

## Conclusion

Presenting public engagement as a key mechanism for advancing health equity discounts the public’s diverse values and priorities. This view rests on the flawed assumption that poor and marginalized people overwhelmingly prioritize health equity over health maximization or other values. Moving forward, policy-makers should promote equity through substantive policy decisions. However, this does not mean that they should reject public engagement. They should employ engagement not as a means to a predetermined end but as a tool of responsive policy-making in two ways: by eliciting public values, and by leveraging the insights of patients regarding service provision.

## References

[1] Open and inclusive: fair processes for financing universal health coverage. Washington, DC: World Bank; 2023. Available from: https://openknowledge.worldbank.org/handle/10986/39953 [cited 2024 Oct 15].

[2] Making fair choices on the path to universal health coverage: final report of the WHO Consultative Group on Equity and Universal Health Coverage. Geneva: World Health Organization; 2014. Available from: https://iris.who.int/bitstream/handle/10665/112671/9789241507158_eng.pdf [cited 2024 Oct 15].

[3] Charlton V. Justice, transparency and the guiding principles of the UK’s National Institute for Health and Care Excellence. Health Care Anal. 2022 Jun 15;30(2):115–45. 10.1007/s10728-021-00444-y 34750743 PMC8575159

[4] Glassman A, Chalkidou K. Priority-setting in health: building institutions for smarter public spending. London: Center for Global Development; 2012. Available from: https://www.cgdev.org/sites/default/files/1426240_file_priority_setting_global_health_FINAL_0.pdf [cited 2024 Feb 15].

[5] Blaauw D, Chambers C, Chirwa T, Duba N, Gwyther L, Hofman K, et al. Introducing an ethics framework for health priority-setting in South Africa on the path to universal health coverage. S Afr Med J. 2022 Mar 2;112(3):240–4. 10.7196/SAMJ.2022.v112i3.16278 35380528

[6] Hunter DJ, Kieslich K, Littlejohns P, Staniszewska S, Tumilty E, Weale A, et al. Public involvement in health priority setting: future challenges for policy, research and society. J Health Organ Manag. 2016 Aug 15;30(5):796–808. 10.1108/JHOM-04-2016-0057 27468775

[7] Kieslich K, Littlejohns P, Weale A. Improving equitable access to health care through increasing patient and public involvement in prioritisation decisions. J Health Organ Manag. 2016 Aug 15;30(5): "https://pubmed.ncbi.nlm.nih.gov/27468625" 10.1108/JHOM-06-2016-0120 27468772

[8] World development report 2004: making services work for poor people. Washington, DC: World Bank; 2004. Available from: https://documents1.worldbank.org/curated/en/832891468338681960/pdf/268950WDR00PUB0ces0work0poor0people.pdf [cited 2024 Oct 15].

[9] Citizen participation and pro-poor budgeting. New York: Department of Economic and Social Affairs; 2020. Available from: https://desapublications.un.org/file/1122/download [cited 2024 Oct 15].

[10] Exploring the role of participatory budgeting in accelerating the SDGs: a multidimensional approach in Escobedo, Mexico. Kenya: United Nations Human Settlements Programme; 2020. Available from: https://unhabitat.org/sites/default/files/2020/08/exploring_the_role_of_participatory_budgeting_and_sdgs_eng.pdf [cited 2024 Oct 15].

[11] Daniels N. Just health: meeting health needs fairly. Cambridge: Cambridge University Press; 2008.

[12] Guttman N, Shalev C, Kaplan G, Abulafia A, Bin-Nun G, Goffer R, et al. What should be given a priority – costly medications for relatively few people or inexpensive ones for many? The Health Parliament public consultation initiative in Israel. Health Expect. 2008 Jun;11(2):177–88. 10.1111/j.1369-7625.2007.00485.x 18429997 PMC5060441

[13] National deliberative poll in Chile [internet]. Participedia; 2020. Available from: https://participedia.net/case/8458 [cited 2024 Oct 15].

[14] McCoy MS, Emanuel EJ. Public engagement in health policy: mapping aims and approaches. In: Lever A, Poama A, editors. The Routledge handbook of ethics and public policy. Abingdon-on-Thames: Routledge; 2018. 10.4324/9781315461731-30

[15] McCoy MS, Warsh J, Rand L, Parker M, Sheehan M. Patient and public involvement: two sides of the same coin or different coins altogether? Bioethics. 2019 Jul;33(6):708–15. 10.1111/bioe.12584 30957902 PMC7116097

